# Supplementary material for: Identification of photoperiod-induced specific miRNAs in the adrenal glands of Sunite sheep (Ovis aries)
Source: Front Vet Sci. 2022 Jul 22;9:888207. doi: 10.3389/fvets.2022.888207 (PMC9354845; doi:10.3389/fvets.2022.888207)
Supplement: Supplementary file 1 [file Data_Sheet_1.ZIP › Supplementary file/miRDeep2 file.zip!/miRDeep2 file/Know_Structure/SPLP42A1/oar-mir-154a.pdf]

miRBase precursor : oar-mir-154a
Total read count : 613
oar-miR-154a-5p read count : 10
oar-miR-154a-3p read count : 602
remaining reads : 1

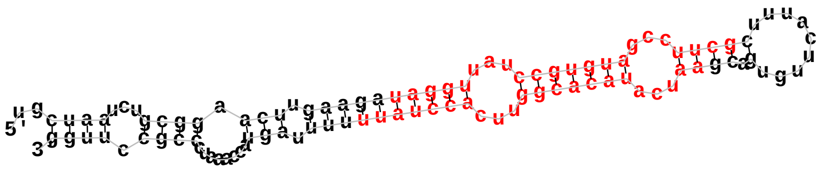

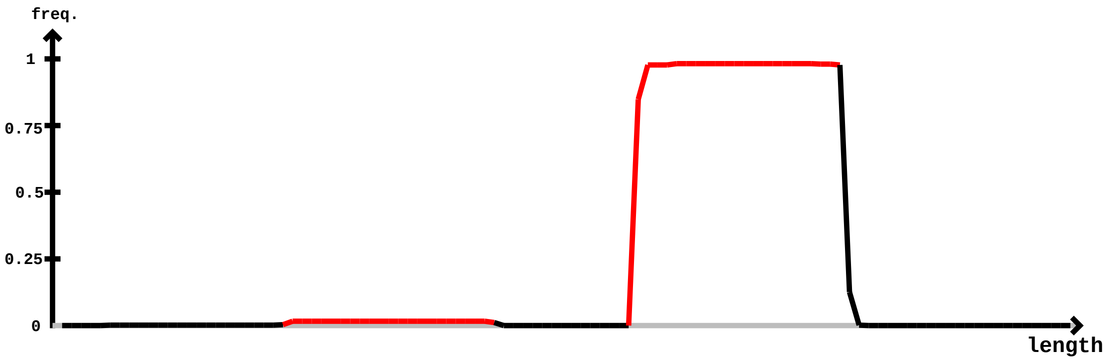


```
                    oar-miR-154a-5p
                                        oar-miR-154a-3p
5'- ugcuaaucugcggaacuugaagauagguuauccguguagccuucgcuuuacuugugacgaaucauacacgguucaccuauuuuuuaguaccaaaucccgccuugg   -3'    exp
    .((......))((.(((.((((((((((...(((((((...(((((.........).))))...)))))))...)))))))))).))).))..............    reads      mm         sample
    ....aaucugcggaacuugaaga..................................................................................    1          0          seq
    ......................auagguuauccguguagccuuc.............................................................    1          0          seq
    .......................uagguuauccguguagccuuc.............................................................    2          0          seq
    .......................uagguuauccguguagccuucg............................................................    7          0          seq
    ...........................................................aaucauacacgguucaccu...........................    1          0          seq
    ...........................................................aaucauacacgguucaccuau.........................    2          0          seq
    ...........................................................aaucauacacgguucaccuauu........................    503        0          seq
    ...........................................................aaucauacacgguucaccuauuu.......................    14         0          seq
    ............................................................aucauacacgguucaccuauu........................    16         0          seq
    ............................................................aucauacacgguucaccuauuu.......................    62         0          seq
    ............................................................aucauacacgguucaccuauuuu......................    1          0          seq
    ...............................................................auacacgguucaccuauu........................    3          0          seq
```
